# Heat shock protein Grp78/BiP/HspA5 binds directly to TDP-43 and mitigates toxicity associated with disease pathology

**DOI:** 10.1038/s41598-022-12191-8

**Published:** 2022-05-17

**Authors:** Liberty François-Moutal, David Donald Scott, Andrew J. Ambrose, Christopher J. Zerio, Marina Rodriguez-Sanchez, Kumara Dissanayake, Danielle G. May, Jacob M. Carlson, Edward Barbieri, Aubin Moutal, Kyle J. Roux, James Shorter, Rajesh Khanna, Sami J. Barmada, Leeanne McGurk, May Khanna

**Affiliations:** 1 Department of Pharmacology, College of Medicine, University of Arizona, Tucson, AZ 85724 USA; 2 Center for Innovation in Brain Science, Tucson, AZ 85721 USA; 3 Pharmacology and Toxicology, School of Pharmacy, University of Arizona, Tucson, AZ 85724 USA; 4 Department of Molecular Pathobiology, NYU, New York, NY USA; 5 Cell and Developmental Biology, School of Life Sciences, University of Dundee, Dow Street, Dundee, DD1 5EH UK; 6 Enabling Technologies Group, Sanford Research, Sioux Falls, SD USA; 7 Department of Biochemistry and Biophysics, Perelman School of Medicine, University of Pennsylvania, Philadelphia, PA 19104 USA; 8 Department of Pediatrics, Sanford School of Medicine, University of South Dakota, Sioux Falls, SD USA; 9 Department of Neurology, University of Michigan, Ann Arbor, MI 48109 USA; 10 Department of Molecular Pathobiology, College of Dentistry, NYU, 433 1st Ave, New York, NY 10010 USA

**Keywords:** Biophysical chemistry, Nucleic acids, Proteins

## Abstract

Amyotrophic lateral sclerosis (ALS) is a fatal neurodegenerative disease with no cure or effective treatment in which TAR DNA Binding Protein of 43 kDa (TDP-43) abnormally accumulates into misfolded protein aggregates in affected neurons. It is widely accepted that protein misfolding and aggregation promotes proteotoxic stress. The molecular chaperones are a primary line of defense against proteotoxic stress, and there has been long-standing interest in understanding the relationship between chaperones and aggregated protein in ALS. Of particular interest are the heat shock protein of 70 kDa (Hsp70) family of chaperones. However, defining which of the 13 human Hsp70 isoforms is critical for ALS has presented many challenges. To gain insight into the specific Hsp70 that modulates TDP-43, we investigated the relationship between TDP-43 and the Hsp70s using proximity-dependent biotin identification (BioID) and discovered several Hsp70 isoforms associated with TDP-43 in the nucleus, raising the possibility of an interaction with native TDP-43. We further found that HspA5 bound specifically to the RNA-binding domain of TDP-43 using recombinantly expressed proteins. Moreover, in a *Drosophila* strain that mimics ALS upon TDP-43 expression, the mRNA levels of the HspA5 homologue (Hsc70.3) were significantly increased. Similarly we observed upregulation of HspA5 in prefrontal cortex neurons from human ALS patients. Finally, overexpression of HspA5 in *Drosophila* rescued TDP-43-induced toxicity, suggesting that upregulation of HspA5 may have a compensatory role in ALS pathobiology.

## Introduction

Proteostasis is the proper equilibrium between the biogenesis, folding, trafficking and degradation of proteins within the cellular milieu^1^. Any interference in proteostasis leads to accumulation of misfolded proteins, a central pathological hallmark of several neurodegenerative diseases including Alzheimer’s disease and amyotrophic lateral sclerosis (ALS)^2,3^. In over 95% of ALS patients, TAR DNA-binding protein of 43 kDa (TDP-43) is mislocalized from the nucleus to the cytoplasm where it misfolds and aggregates in affected neurons and glia^1,2^. Several fragments from the C-terminal region of TDP-43, traditionally referred to as CTFs, have been detected in post-mortem tissue from  patients with TDP-43 proteinopathies^4–6^, but their exact nature and abundance seem to vary between tissues, patients and/or mode of detection^7^. TDP-43 pathology has been observed across several neurodegenerative disorders including frontotemporal degeneration (FTD), Alzheimer’s disease, and limbic-predominant age-related TDP-43 encephalopathy (LATE)^5,8–10^. Although the causative factors that lead to TDP-43 aggregation are still not fully understood, studies implicate proteostasis mechanisms such as impaired autophagy and the ubiquitin proteasome system (UPS)^11,12^ as well as compromised endolysosomal function^13–15^. TDP-43, a DNA/RNA-binding protein, consists of a folded N-terminal domain (NTD) linked by a flexible loop to two tandem RNA recognition motifs (RRMs)—RRM1 and RRM2—and a predominantly unfolded C-terminal prion-like domain that harbors the majority of disease-associated mutations in ALS^16^. TDP-43 functions primarily in RNA metabolism including splicing, translation and the cytoplasmic stress granule response^17^. Thus, in ALS, TDP-43 aggregation leads to repression of TDP-43-controlled pathways as well as a dysregulation of proteostasis^18,19^.

Central to proteostasis are the chaperones; a large family of proteins that typically bind to exposed hydrophobic sequences to assist in protein misfolding, degradation, and the clearance of aggregated protein^20,21^. One major chaperone subfamily is the evolutionarily conserved Hsp70s, which consists of 13 gene products (HspA1A, HspA1B, HspA1L, HspA2, HspA5, HspA6, HspA7, HspA8, HspA9, Hsp12A, Hsp12B, Hsp13 and Hsp14))^22,23^. The canonical Hsp70 proteins share high sequence identity and have diverse cellular localizations and functions^22^. All canonical Hsp70 proteins have an N-terminal nucleotide binding domain (NBD) and a C-terminal substrate-binding domain (SBD) that allosterically communicate in an ATP-dependent manner to recognize and bind client proteins^24^.

Typically, high levels of Hsp70 can be produced by cells in response to hyperthermia, oxidative stress, changes in pH, chemical disruption of proteostasis^25^ and expression of disordered proteins^26–28^. Intriguingly, in motor neurons, the primary cells affected in ALS, there appears to be an incomplete stress response, as inferred from the lack of Hsp70 upregulation in response to several stress paradigms^29,30^. Moreover, overexpression of chaperones, including Hsp70s, prevented TDP-43 aggregate formation, more specifically CTF-25 (or TDP-25, a 25 kDa C-terminal fragment of TDP-43) aggregation^31^ and injection of recombinant human Hsp70 was effective in improving motor defects as well as increasing lifespan of a superoxide dismutase type 1 (SOD1) mouse model of ALS^32^. Collectively, these findings may partially explain why strategies to boost Hsp70 have been touted as neuroprotective in neurodegenerative diseases, particularly ALS. In support of this, Arimoclomol, a co-inducer of heat shock protein expression, has been under investigation in a clinical trial for ALS patients but recently failed in phase II/III (Clinicaltrials.gov identifier NCT03491462). Arimoclomol is known to prolong heat shock factor 1 (HSF1) binding to the heat shock element (HSE) localized in the promoter of inducible Hsp70 isoforms, and it induces expression of a certain subset of heat shock proteins in neuronal cell lines^33^. As not all Hsp70s are controlled by the HSE, this might indicate that only a precise Hsp70 isoform subset is able to mitigate ALS toxicity.

It is still unclear how and which Hsp70 isoforms regulate TDP-43. Previous studies demonstrate that at least three Hsp70 isoforms immunoprecipitate with TDP-43: HspA1A, HspA5 and HspA8^34^. It was later hypothesized that Hsp70s could be constitutively bound to TDP-43. Upon a heat shock event, Hsp70 could be released from its interaction with TDP-43 as misfolded proteins accumulate, which could thereby promote the formation of TDP-43 aggregates^35^. More recently, it was shown that in cells, several Hsp70 isoforms accumulate within mutated TDP-43 phase separated anisosomes (an anisotropic intranuclear liquid spherical shell)^36^. To date, potential direct binding between the Hsp70 isoforms and TDP-43 has not been investigated. Here, we interrogated the association of TDP-43 with specific Hsp70 isoforms using BioID, a technique that leverages the activity of a promiscuous biotin ligase to biotinylate proteins based on proximity^37^. We found that HspA5 and HspA8 were enriched in the nuclear, but not cytoplasmic, fraction of TDP-43. We further tested direct binding of TDP-43 with the Hsp70 isoforms HspA1A, HspA5 and HspA8 and found that the TDP-43 RRM domains selectively bind HspA5. Moreover, the mRNA levels of the HspA5 homologue (Hsc70.3) in *Drosophila melanogaster* (*Drosophila*) were significantly increased upon TDP-43 expression and we observed an upregulation of HspA5 in prefrontal cortex neurons of human ALS patients. Finally, we discovered that upregulation of Hsc70.3 in *Drosophila* protects against TDP-43-induced toxicity while the ATP binding-deficient mutant Hsc70.3^K97S^ variant^35^ had no effect. Our data underscore an Hsp70 isoform preference by TDP-43 and thus position induction of HspA5 binding to TDP-43 as a novel therapeutic strategy for mitigating TDP-43 toxicity.

## Results

### BioID identifies Hsp70 networks binding to TDP-43 in the nucleus

**Figure 1:**
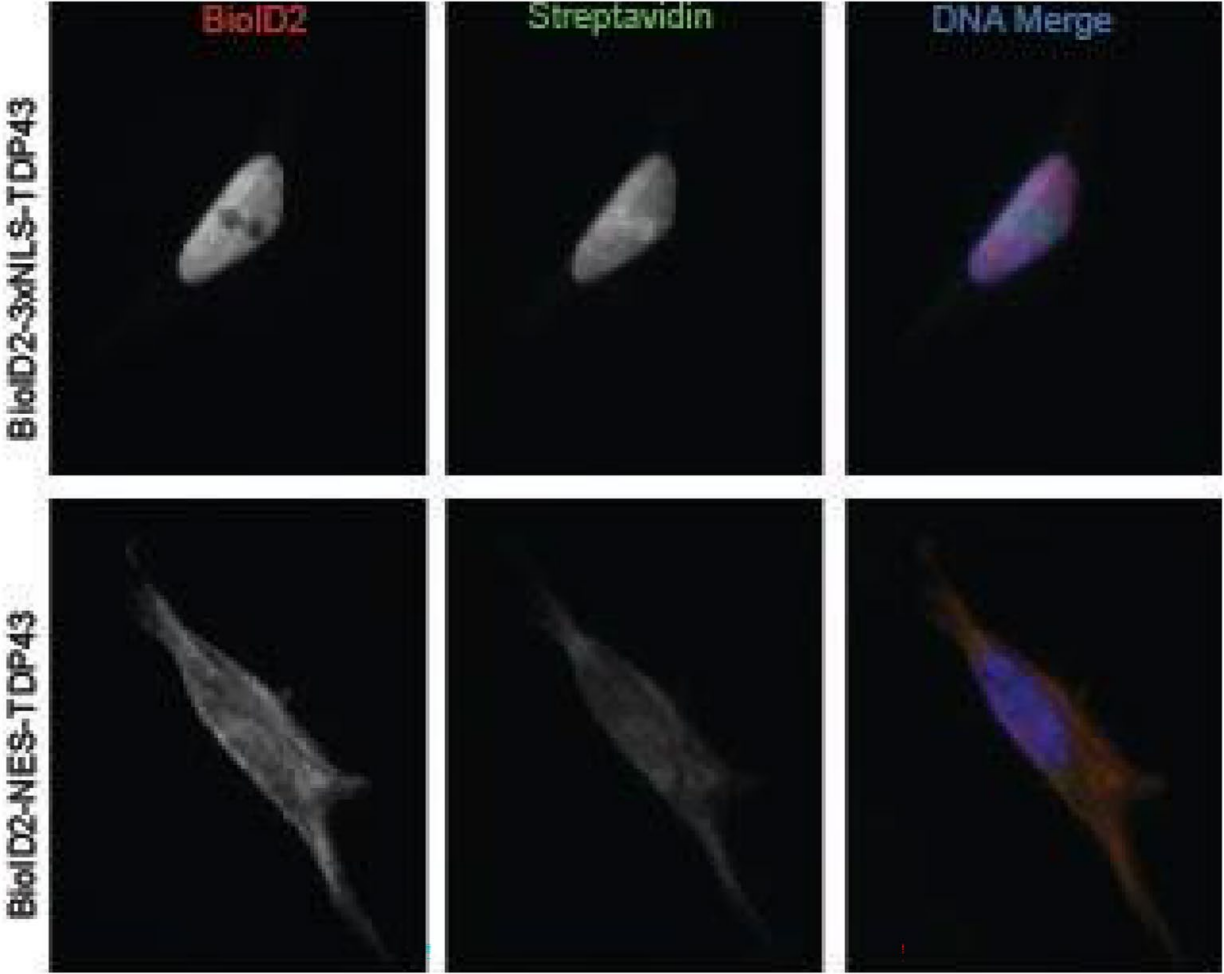
BioID of TDP-43 in SH-SY5Y cells in the nucleus and cytoplasm. Epifluorescence images for validating fusion-protein (*red*) expression and promiscuous biotinylation (*green*) localization following the addition of biotin.

To characterize nuclear versus cytoplasmic localization as well as possible Hsp70 isoform specificity of TDP-43, we performed proximity-dependent biotin labeling (BioID) of TDP-43 in the nucleus or the cytoplasm. BioID2 was fused to the N-terminal domain of TDP-43, and either a 3 × tandem nuclear localization signal (3xNLS) or a nuclear export signal (NES) was added to localize TDP-43 to the nucleus or cytoplasm, respectively. BioID2-3xNLS-TDP43, BioID2-NES-TDP43 or the BioID2 control were stably expressed in human neuroblastoma SH-SY5Y cells, and its localization was verified using immunofluorescence (Fig. 1). It is worth noting that while BioID2-NES-TDP-43 mostly localized to the cytoplasm, some marginal nuclear localization was observed and is likely due to the intrinsic NLS of TDP-43. Cells expressing each TDP-43 variant or control vector were lysed for BioID pulldown in triplicate, and affinity capture of biotinylated proteins was confirmed via western blot (Fig. S1). Biotinylated proteins identified via mass spectrometry (MS) were ranked by label-free quantification (LFQ) intensity and enrichment compared to control, and the number of replicates (N) of each protein was identified. Following a criterion of threefold enrichment over control and N ≥ 2 threshold, 144 nuclear and 28 cytoplasmic interaction candidates for TDP-43 were identified (Table S1). “Highest confidence associations” were proteins found only in the BioID2-3xNLS-TDP43 or BioID2-NES-TDP43 samples, and not at all in the control BioID samples, ranked by LFQ intensity. “Good confidence associations” were proteins enriched at least threefold over control, ranked by experimental:control intensity ratio.

Surprisingly, HspA5 and HspA8 were found as highest confidence and good confidence associations respectively in the nuclear TDP-43 sample (BioID2-3xNLS-TDP-43) (Table S1). No Hsp70 isoform was identified in the cytoplasmic TDP-43 sample (BioID2-NES-TDP43), suggesting an absence of such an interaction with TDP-43 in the cytoplasm without stress (Table S1). HspA8 is well described for its implication in nuclear import of client proteins as it shuttles between the cytoplasm and nucleus^38^. Although HspA5 is mostly known for its ER localization, several studies have shown the presence of HspA5 in the nucleus^36,39,40^, including in SH-SY5Y cells^41^. Thus, our data suggest that in the SH-SY5Y cells and in the absence of stress, HspA5 and HspA8 selectively associate with nuclear but not cytoplasmic, TDP-43.

### The RRM domains of TDP-43 selectively bind to the Hsp70 isoform HspA5

The BioID data hinted towards TDP-43 binding selectively to Hsp70 isoforms, as demonstrated by the fact that only HspA8 and HspA5 were found to be significantly enriched. Another previous study showed via immunoprecipitation that Hsp70 interacts with TDP-43 primarily through its RRMs^33^, but the exact Hsp70/TDP-43 interface was never investigated. We thus set out to characterize the binding of TDP-43 to different Hsp70 isoforms. To this end, we selected HspA5 and HspA8 (identified from BioID) and HspA1A, an Hsp70 isoform implicated in TDP-43 binding^34^.

We first predicted where Hsp70 could bind to TDP-43 using LIMBO, a position specific algorithm for identifying Hsp70 binding sites in proteins^42^. LIMBO is based on a position-specific scoring matrix (PSSM) trained from in vitro peptide binding data and structural modelling and predicts the binding of bacterial Hsp70 homolog DnaK, which shares ~ 50% identity with human Hsp70 isoforms. For Hsp70 prediction, TDP-43 was divided into three fragments: aa 1–120 (N-terminal domain and flexible linker of TDP-43 (NTD)), aa 101–269 (the two RNA recognition motifs (RRM)) and aa 270–414 (C-terminal prion-like domain, which is mostly unstructured, aggregation prone, and the site for most ALS mutations) (Fig. S2A). While predicted binding sites were noted in the NTD and RRM domains, the algorithm did not predict any Hsp70 binding sites in the C-terminal prion-like domain (Fig. S2B,C). Thus, our computational predictions suggest that Hsp70 does not bind to the unstructured C-terminal domain, but does bind the NTD and RRMs.

Using microscale thermophoresis (MST), we measured the binding of the substrate binding domain (SBD) of HspA1A, HspA5 and HspA8 to TDP-43_1–102_, a construct corresponding to TDP-43-NTD. The SBD of these Hsp70 isoforms is approximately 200 amino acids long and is composed of a two layered twisted β-sheet and a C-terminal α-helical subdomain. The SBD and its binding to the client peptide are allosterically modulated by the ATP binding site. However, binding of ATP to the TDP-43 RRM domains has also been shown to enhance the stability of TDP-43^43^. Thus, we reasoned that this may inhibit Hsp70 isoform binding, and opted to use an Hsp70 construct that lacked the N-terminal nucleotide binding site but retained the ability to recognize client peptides.

**Figure 2:**
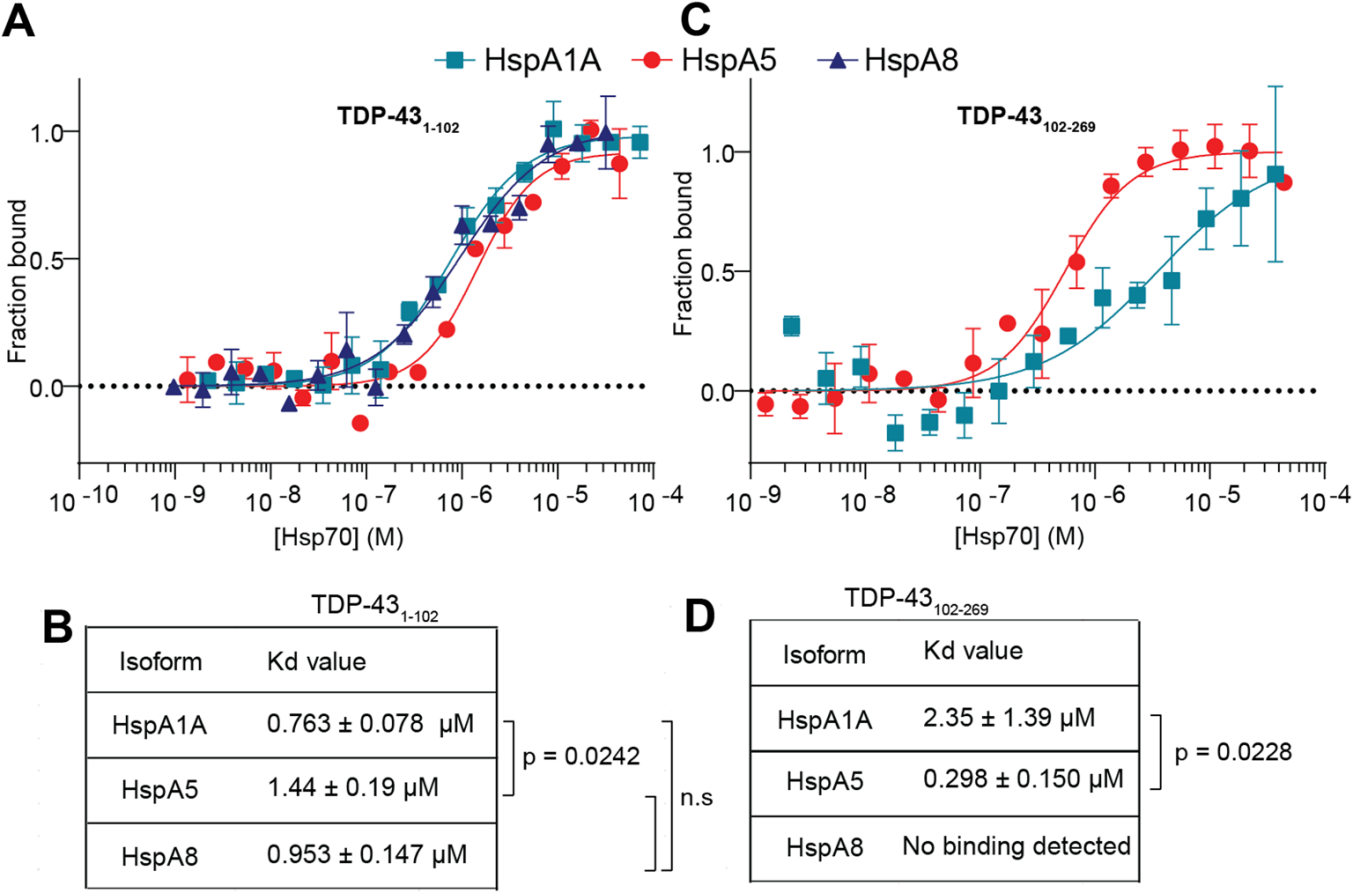
Selective binding of native TDP-43 constructs to Hsp70 isoforms. Microscale thermophoresis was used to measure the binding of ranging concentrations of Hsp70 SBDs to 50 nM of labelled TDP-43_1–102_ (**A**) or TDP-43_102–269_ (**C**). HspA1A and HspA5 were able to bind TDP-43_102–269_. No signal was detected for HspA8 binding to TDP-43_102–269_. (**B**) Table of affinity constants extracted from the MST values for TDP-43_1–102_. (**D**) Table of affinity constants extracted from the MST values for TDP-43_102–269_. Statistical difference was assessed between HspA1A and HspA5 binding (Welch’s test). Data is presented as Mean ± SD (n = 3). Statistical difference was assessed between HspA1A, HspA5 and HspA8 binding (Brown-Forsythe and Welch ANOVA test).

All three Hsp70 isoforms bound TDP-43_1–102_ with a similar affinity calculated to be in the high nanomolar to low micromolar range (Fig. 2A,B). There was a small but significant difference in the binding affinity between the between binding of HspA1A and HspA5 to TDP-43_1–102_ (p = 0.0242, Fig. 2B). By contrast, in MST experiments with the RRM domain (TDP-43_102–269_), we found that HspA8 did not bind at all, and that HspA5 (298 ± 150 nM) bound with greater affinity than HspA1A (2.35 ± 1.39 μM) (Fig. 2C,D). TDP-43_102–269_ binding to HspA5 exhibited a significantly lower Kd than HspA1A (p = 0.0228; Fig. 2D), indicating that TDP-43_102–269_ was selective for HspA5 over the other isoforms tested. Overall, our data indicate that while the unstructured NTD of TDP-43 binds to HspA1A, HspA5 and HspA8 with almost equal affinity; the conformationally stable RRM domains of TDP-43 have greatest propensity to bind HspA5.

### HspA5 binds TDP-43 RRM2 at the interface with RNA

**Figure 3:**
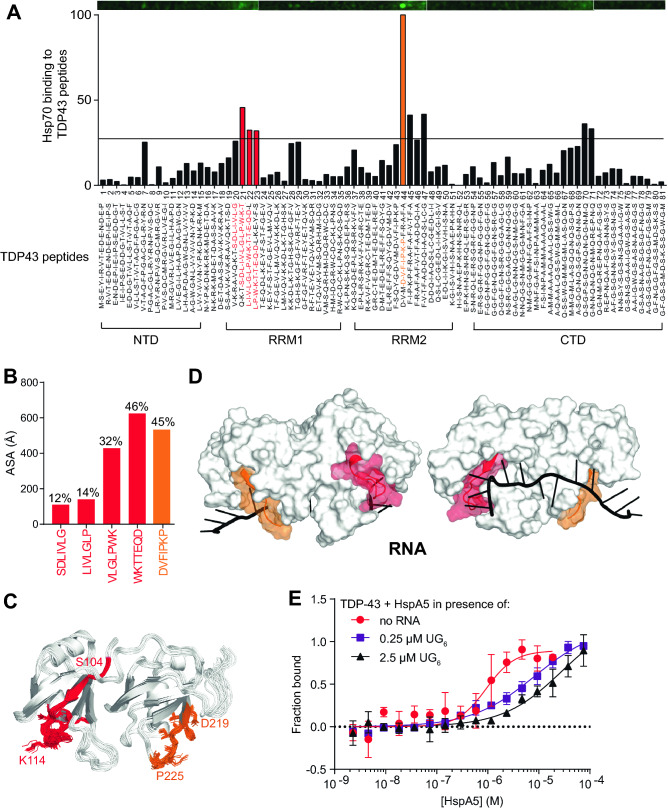
Mapping of the Hsp70 client peptides to the RRM and RNA-binding interface of TDP-43. (**A**) Binding of HspA5 on immobilized 15-mer TDP-43 peptides, in overlapping five amino acid steps. The blots were scanned, and spot intensities were quantified and represented as a normalized signal. Highly scored LIMBO predicted client peptides of TDP-43 are shown in red and orange, respectively. (**B**) Accessibility of the client peptides from the peptide array at the TDP-43 surface (ASA) were calculated using Areaimol as implemented in the CCP4 suite^35^ on the free form structure of the tethered RRM domains (PDB ID 4bs2^22^). To note, there is no significant difference in accessibility values when RNA is present. The percentage of accessibility represents the ASA of the motif compared to the total surface of TDP-43. (**C,D**) Mapping of the client peptides on TDP-43 or NMR structures (cartoon representation) (**C**) or surface (**D**) of the RRM domain (PDB code: 4bs2^22^). The predicted client peptides were color coded as described in (**A**). (**E**) Microscale thermophoresis of NTA-labelled TDP-43_102–269_ interaction with HspA5 in the absence or in the presence of increasing concentrations of UG_6_ RNA. The presence of RNA shifted the Kd of the TDP-43_102–269_/HspA5 interaction from 0.89 ± 0.25 µM (*red* curve) to 28.3 ± 23.7 µM (*black* curve). Data is presented as Mean ± SD (n = 3).

Spurred by the selective binding of the RRM region of TDP-43 (TDP-43_109–260_) to HspA5, we set out to experimentally map, in greater resolution, potential HspA5 binding sites within TDP-43. To do this we synthesized a peptide array of 15-mer peptides with an overlap of 5 amino acids that spanned the RRM region of TDP-43. The peptide array was incubated with HspA5-SBD protein and peptide binding was detected using an antibody directed against HspA5 (Fig. 3A). HspA5 bound to several TDP-43 peptides in RRM1 (noted in red in Fig. 3A) and in RRM2 (highest binding peptide shown in orange in Fig. 3A). Some C-terminal TDP-43 peptides also bound to HspA5, but this could be due to the fact that these C-terminal peptides (e.g., peptide 70) have several glutamine (Q) and asparagine (N) amino acids, typical of prion-like domains. There was positive concordance between our computationally predicted sites (Fig. S2B,C) and peptides in the RRM1 and RRM2 domains of TDP-43 that bound HspA5.

We next mapped these potential HspA5-binding regions on TDP-43_102–269_ in the context of the 3-dimensional and folded structure of TDP-43. We calculated the surface accessibility of the TDP-43 peptides bound by Hsp70 and mapped the peptide sequence on to the known TDP-43 structures of the RRM domains complexed to (UG)_6_ RNA (PDB code: 4BS2^44^) (Fig. 3). Notably, all of the TDP-43 peptides bound by HspA5 in the NTD and RRM domains have partial surface accessibility (Fig. 3B). Moreover, they have relatively low dynamics in the NMR structures and include secondary structural elements (helix for the accessible peptide in RRM1, strand for the accessible peptide in RRM2) (Fig. 3C). Given that HspA5 binds TDP-43_102–269_, these data suggest that (i) HspA5 might recognize only a portion of the peptide, sufficient for initiating binding, and (ii) there might be structural elements at play in the HspA5/TDP-43 interaction.

Interestingly, one TDP-43 client peptide (shown as red in Fig. 3) overlaps with the ribonucleic protein motif-2 (LIVLGL in RRM1). We tested if RNA had an effect on binding using increasing concentrations of UG_6_ RNA, the canonical binding sequence of TDP-43^45^, and we detected a decreased affinity of the HspA5/TDP-43_102–269_ interaction from 0.9 ± 0.3 µM to 28.3 ± 23.7 µM (Fig. 3E). RNA binding is thought to maintain TDP-43 in a soluble state, and has been proposed to prevent passive exit from the nucleus^46,47^. A second client peptide (shown as red in Fig. 3) is adjacent to amyloidogenic sequences in RRM2 (246-EDLIIKGISV-255; shown in orange in Fig. 3)^48^. Since exposed E246/D247 residues are markers of misfolded TDP-43^49^, exposing client peptides following loss of nucleic acid binding and/or exposure of amyloidogenic regions, might trigger HspA5 association to prevent aggregation of TDP-43.

### Upregulation of the HspA5 Drosophila homolog mitigates TDP-43 disease-associated toxicity

**Figure 4:**
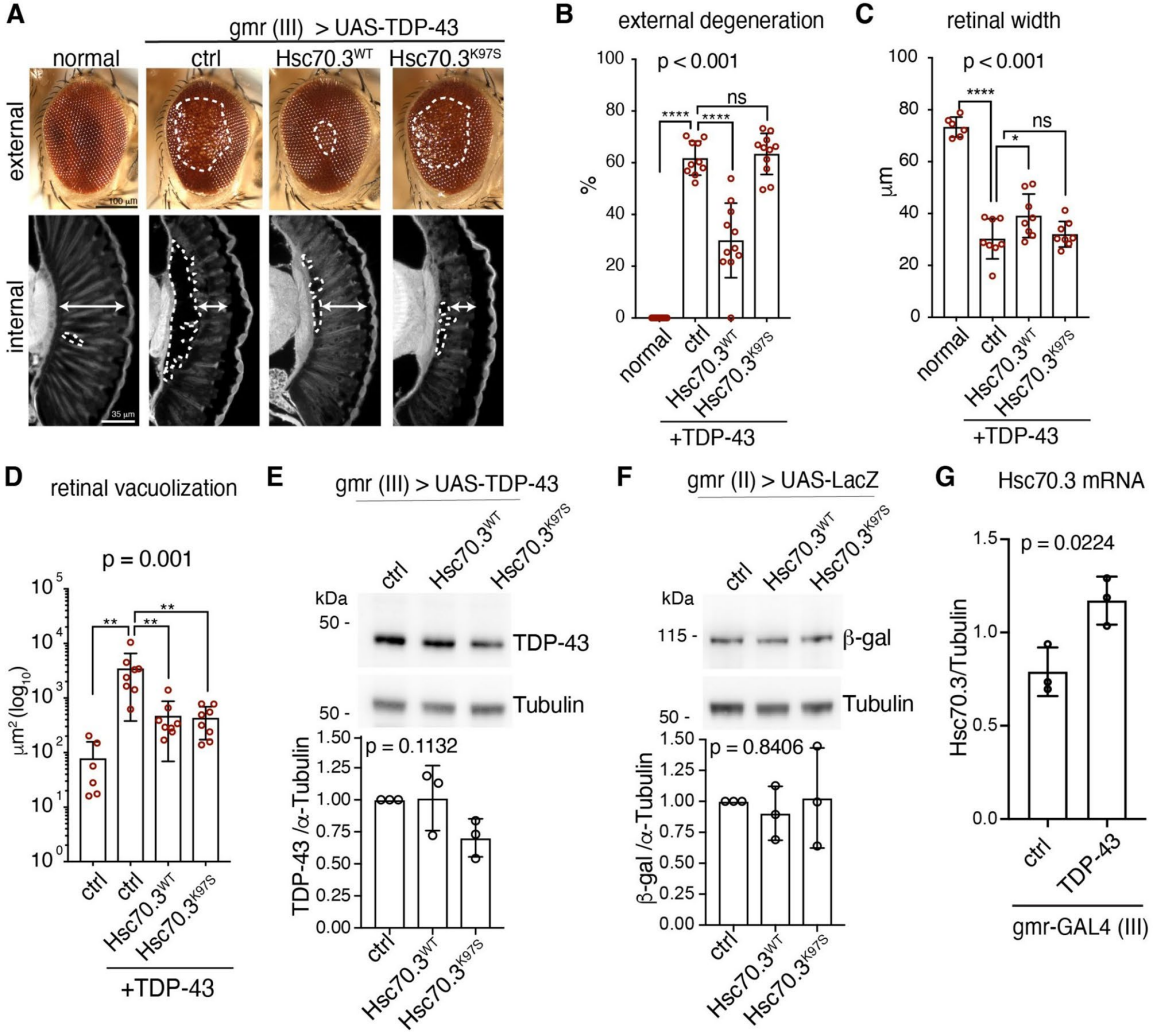
Upregulation of Hsc70.3 mitigates TDP-43-induced toxicity in the Drosophila eye. (**A**) Compared to the normal control, expression of human TDP-43 (ctrl) in the Drosophila eye disrupts the external eye (white hatched line, top panel) and internal retina (white double headed arrow, and white hatched line lower panel). (**B**) Expression of Hsc70.3 suppresses TDP-43-induced disruption of the external eye. Data is presented as Mean ± SD, one way ANOVA and a Fisher’s LSD test. ****P < 0.0001 and *ns* not significant. (**C**) Expression of Hsc70.3^WT^ suppresses TDP-43-induced reduction of retinal width (see double headed arrow, lower panel in A). Data is presented as Mean ± SD, one way ANOVA and a Fisher’s LSD test. ****P < 0.0001, *P < 0.05 and *ns* not significant. (**D**) Expression of Hsc70.3^WT^, and Hsc70.3^K97S^ reduces TDP-43-induced vacuolization of the internal eye (white hatched line, lower panel in A.). Data is presented as Mean ± SD, one way ANOVA and a Fisher’s LSD test. ****P < 0.0001, *P < 0.05 and *ns* not significant. (**E**) Upregulation of Hsc70.3^WT^ or Hsc70.3^K97S^ had no effect of the total protein levels of TDP-43. Protein isolated from ~ 5 to 10 male heads immunoblotted for TDP-43 and Tubulin. Protein levels were quantified from 3 independent biological repeats. Data is presented as Mean ± SD, one-way ANOVA and Tukey’s test, *ns* not significant. (**F**) Upregulation of Hsc70.3^WT^ or Hsc70.3-^K97S^ had no effect of the total protein levels of β-galactosidase. Protein isolated from ~ 5 to 10 male heads immunoblotted for β-galactosidase and Tubulin. Protein levels were quantified from 3 independent biological repeats. Data is presented as Mean ± SD, one-way ANOVA and Tukey’s test, ns: not significant. (**G**) Expression of TDP-43 with gmr-GAL4 leads to an increase in Hsc70.3 mRNA levels compared to control (ctrl). Data is presented as Mean ± SD. An unpaired and two-tailed T test was used to determine significance. Genotypes are (**A–E**) normal is *y, sc, v, sev/w*^*1118*^*;* +*/*+*; gmr-GAL4 (YH3)/si.mCherry*^*35783*^, ctrl is *y, sc, v, sev/w*^*1118*^*; UAS-TDP-43/*+*; gmr-GAL4 (YH3)/ si.mCherry*^*35783*^, Hsc70.3^WT^ is *w-; UAS-TDP-43/ UAS-Hsc70-3*^*WT*^*; gmr-GAL4 (YH3)/*+ and Hsc70.3^K97S^ is *w-; UAS-TDP-43/UAS- Hsc70-3*^*K97S*^*; gmr-GAL4 (YH3)/*+. (**F**) ctrl is *y, sc, v, sev/w*^*1118*^*; UAS-LacZ/*+*; gmr-GAL4 (YH3)/si.mCherry*^*35783*^, Hsc70.3^WT^ is *w−; UAS-LacZ/UAS-Hsc70-3*^*WT*^*; gmr-GAL4 (YH3)/*+ and Hsc70.3^K97S^ is *w-; UAS-LacZ/UAS-Hsc70-3*^*K97S*^*; gmr-GAL4 (YH3)*.

Since we found a direct and specific interaction of HspA5 with native TDP-43, we set out to determine if HspA5 could modulate TDP-43 toxicity using a *Drosophila melanogaster* (Drosophila) model of TDP-43 toxicity^50,51^. Compared to the expression of a normal control (si.mCherry), expression of human TDP-43 in the Drosophila eye disrupts the external surface (compare 0% to 61.7% ± 6.9%, normal vs TDP-43 respectively (Fig. 4A,B)), reduces retinal width (compare 73.3 ± 3.8 μm to 30.3 ± 7.7 μm, normal vs TDP-43 respectively (Fig. 4A,C)) and causes retinal vacuolization (compare 78.1 μm^2^ to 3469 μm^2^, normal vs TDP-43 respectively (Fig. 4A–D))—all of which are readouts of TDP-43-associated toxicity.

The Drosophila HspA5 homologue is Hsc70.3 (Fig. S3A,B). In the absence of TDP-43, upregulation of the normal form of Hsc70.3 (Hsc70.3^WT^) had no effect on the Drosophila eye. However, by contrast, downregulation of Hsc70.3 altered the structure of the external eye indicating that loss of Hsc70.3 is detrimental to the Drosophila eye (Fig. S3C). We thus focused on the effect of upregulating Hsc70.3 on TDP-43 toxicity. Co-expression of Hsc70.3^WT^ with TDP-43 in the Drosophila eye significantly improved the disruption to the external and internal eye morphology induced by TDP-43 (Fig. 4A–D). To address whether the suppression of TDP-43 toxicity by Hsp70.3 upregulation required ATPase activity, we expressed Hsc70.3 variants with defective ATPase activity (Hsc70.3^D31S^ and Hsc70.3^K97S^)^35^ in the Drosophila eye and selected the variant that conferred no toxicity ((Hsc70.3^K97S^, (Fig. S3C)). Co-expression of Hsc70.3^K97S^ with TDP-43 had no effect on TDP-43-induced toxicity of the external eye and retinal width but suppressed TDP-43-induced vacuolization (Fig. 4A–D), indicating that Hsc70.3 requires ATPase activity to fully suppress TDP-43 toxicity. Importantly, upregulation of Hsc70.3^WT^ or Hsc70.3^K97S^ had no effect on the total protein levels of TDP-43 or a control protein (β-galactosidase) (Fig. 4E,F, Fig S4), indicating that the suppression of TDP-43 by Hsc70.3 is not simply because of reduced TDP-43 protein levels. Collectively, our data indicate that upregulation of Hsc70.3 is beneficial in preventing TDP-43-associated toxicity in *Drosophila*.

Considering the interaction between TDP-43 and HspA5, as well as the mislocalization of TDP-43 in ALS, we next set out to determine if in our Drosophila model of TDP-43 disease Hsc70.3 levels were upregulated. Due to the lack of antibodies available to Hsc70.3 we opted to measure the levels of Hsc70.3 mRNA in Drosophila expressing either a normal control (si.mCherry) compared to Drosophila expressing TDP-43. This revealed that Hsc70.3 mRNA levels, relative to Tubulin, were significantly increased upon TDP-43 expression ((compare 0.79 ± 0.13 (SD) to 1.17 ± 0.13 (SD), control vs TDP-43, respectively) (Fig. 4G). This suggests that the suppression of TDP-43 toxicity observed upon upregulation of Hsc70.3 (Fig. 4A–D) is not due to restoration of reduced Hsc70.3 mRNA levels. Rather, they suggest that upon TDP-43-induced toxicity Hsc70.3 expression is increased, similar to what we seem to observe in human ALS postmortem tissue (Fig. S5). It is enticing to speculate that Hsc70.3 is upregulated to prevent TDP-43-induced toxicity, but the levels are not sufficient to fully protect the tissue. Thus, boosting Hsc70.3/HspA5 expression levels in ALS patients may be a potential therapeutic strategy.

## Discussion

Targeting the molecular chaperone pathway is a potential therapeutic strategy in neurodegenerative disorders such as ALS. Arimoclomol, a compound that increases Hsp70 proteins as well as other Hsp chaperones^52^, recently failed phase II/III clinical trials for the treatment of ALS (Clinicaltrials.gov identifier NCT03491462). A greater understanding of which HSP family members control TDP-43 will be crucial for insights into the mechanisms that propagate disease as well as in developing more nuanced therapeutic strategies. Here we show that the Hsp70 isoform HspA5 specifically binds to the RNA-binding domain of TDP-43, that there is an apparent increased expression of cytoplasmic HspA5 in the prefrontal cortex of ALS patients and that upregulation of the HspA5 homologue mitigates TDP-43-induced toxicity in Drosophila, identifying HspA5 as a potential target in TDP-43-associated disease.

Although a plethora of proteins and protein families have been reported to interact with and control TDP-43, they have generally been identified using indirect measures such as genetic interaction screens^50,51,53–58^ and affinity pull-down methods^34,57,59,60^. Here, we used BioID, a unique technique that leverages promiscuous nature of biotin ligase to biotinylate proteins based on proximity^37^, on a dividing neuroblastoma cell population (SH-SY5Y cells) expressing TDP-43 to which we added either an extra NES (Nuclear Export Signal) or an NLS (Nuclear Localization signal). We have no information on normal folding as well as sub-nuclear/cytoplasmic localization of such TDP-43 constructs. While our BioID data was able to reproduce, to some extent, several TDP-43 interactions with known partners including HspA8 and HspA5, ribosomal proteins, and other proteins of RNA metabolism^34,59^, the cell type used, and the expression of modified TDP-43 could have impacted the binding partners that were found. Nevertheless, our data indicate that HspA5 and HspA8 were found to bind TDP-43 in the nucleus and in the absence of exogenous stress. HspA5 and HspA8 are constitutively expressed, contrary to HspA1A expression, for example, that is only induced by different stressors. Moreover, even though both HspA5 and HspA8 are commonly known to reside within the endoplasmic reticulum and the cytoplasm respectively, HspA5 is actively translocated to other cellular locations, including mitochondria and the nucleus^41,61,62^, and cytoplasmic HspA8 shuttles between cytoplasm and nucleus, which enables it to import client proteins into the nucleus^63^. Moreover, several Hsp70 isoforms, including HspA5 and HspA8, were found accumulated within mutated TDP-43 phase separated anisosomes (anisotropic intranuclear liquid spherical shells)^36^. Overall, this supports the possibility of a TDP-43/Hsp70 isoform interaction in the nucleus, and while an interaction with Hsp70 chaperones in the cytoplasm was not observed here it is possible that such an interaction may happen upon activation of the stress response.

Using in vitro binding approaches, we further established that HspA1A, HspA5 and HspA8 bind directly to TDP-43. Our data indicate that while the Hsp70 isoforms HspA1A, HspA5 and HspA8 bind to the partially or fully unfolded N-terminal domain of TDP-43 with equal affinities, binding to the conformationally stable RRM domains of TDP-43 is highly selective for HspA5. Using a peptide-binding array we identified HspA5 binding sites in each RRM of TDP-43. The HspA5 binding regions in the RRMs are only partially exposed, which is surprising since chaperones typically recognize hydrophobic stretches of amino acids in unfolded proteins^20^. It is thus possible that the recognition of the RRM domains of TDP-43 by HspA5 needs structural elements in addition to the predicted Hsp70 binding sites and perhaps may be involved in an alternative function to chaperone activity. In the absence of stress, HspA5 maintains the three transmembrane UPR sensors (PERK, IRE1 and ATF6) in an inactive state through direct binding to the respective proteins^64^. Upon ER stress, accumulated misfolded proteins titrate HspA5 away from PERK/IRE1/ATF6, leading to their activation and subsequent stimulation of the UPR^65^. Here, we show that HspA5 binding to TDP-43 is inhibited by RNA. The importance of RNA binding to TDP-43 in maintaining TDP-43 solubility has been previously reported^66,67^, and our data suggest that HspA5 may recognize the non-RNA bound version of TDP-43 to ensure proper folding and/or prevent misfolding or to trap TDP-43, similarly to HspA5 binding to UPR sensors. Another interesting client peptide is 246-EDLIIKGISV-255, encompassing E246 and D247 residues, which exposition is a marker of misfolded TDP-43, as well as a cleavage site generating TDP-43 CTF^68^. In line with this, Hsp70 overexpression prevented TDP-43 aggregate formation of CTF-25 but was unable to disassemble or solubilize those inclusions^31^. However, it is important to note that TDP-43 CTFs may not be imperative for neurodegeneration^69,70^ since studies have detected much less TDP-43 CTFs than the entire protein in ALS spinal cords^6,71,72^.

Expression of TDP-43 in the eye during development leads to adult Drosophila with a disrupted external eye and vacuolization and loss of tissue in the retina. Our data indicate that TDP-43 expression in the developing eye recapitulates HspA5 pathology observed in human ALS as we observe an upregulation of Hsc70.3 mRNA. Furthermore, upregulation of Hsc70.3 mitigates the toxicity of TDP-43 when expressed in the developing eye, implicating upregulation of Hsc70.3/HspA5 as a potential therapeutic strategy. Further studies are needed to address how Hsc70.3 upregulation may mitigate TDP-43 toxicity in aging adult neurons. HspA5 has also been implicated in regulating the toxicity and aggregation of the ALS-causing protein superoxide dismutase (SOD1). For example, knock-in mice expressing HspA5 that lacks the ER retention signal, KDEL, display age-related motor problems, loss of motoneurons and aggregation of wild-type SOD1^73^. Moreover, the neuronal pathology caused by expression of mutant SOD1 (SOD1-G93A) was exacerbated in mice deficient in the HspA5 co-factor SIL1, while SIL1 overexpression induced significant neuroprotection related to improved ER proteostasis and reduced SOD1 aggregation^74^. It is worth noting that previous work showed that in Drosophila down regulation of tankyrase 1 and tankyrase 2 (Tnks-1/2), which physically interact with TDP-43, reduces TDP-43 toxicity while their upregulation enhances TDP-43 toxicity^62,75^. This further suggests that not all binding partners of TDP-43, when upregulated, ameliorate TDP-43 toxicity.

HspA5 mainly localizes to the endoplasmic reticulum (ER) where it controls protein folding during ER-associated stress^76^ but cellular stimuli such as ER stress and ER-associated degradation can lead to the localization of HspA5 to the mitochondria and the cytosol^61,77^. Our data indicates a nuclear interaction between TDP-43 and HspA5 but, conversely, HspA5 was largely localized to the cytoplasm in ALS and aged matched control patients (Fig. S5). While we cannot overlook that our BioID data may have missed interactions as discussed above, or that the cell model used is quite distinct from a post mitotic neuron or a glial cell, cytoplasmic HspA5 could also be explained by defects in nuclear import that occurs with age^78^ as well as neurodegeneration^79^. Indeed, TDP-43 pathology, including aggregates and mutated proteins, are known to sequester transport factors and impact nuclear transport^59^.

Finally, it is worth noting that Arimoclomol is a co-activator that prolongs the binding of activated HSF1 to heat shock elements in the promoter region of many chaperones including Hsp70 family members. Notably, HspA5 expression is not under the control of Hsf1^23,80–82^. Moreover, Arimoclomol, was shown to induce the expression of only HspA6 and HspA1A in human SH-SY5Y cells^33^. The failure of Arimoclomol in phase II/III of clinical trial for ALS patients might be partly explained by a lack of specific Hsp70 isoform targeting.

Overall, the observations in this study suggest that upregulation of HspA5 in ALS may have a compensatory role, prolonging the survival of neurons by preventing TDP-43 misfolding and subsequent toxicity. Elucidating the stimuli and the underlying cellular mechanisms that control HspA5 binding to TDP-43 will provide the platform for investigating HspA5 as a potential therapeutic target in TDP-43-associated disease.

### Materials

All reagents were purchased from Sigma (St. Louis, MO, USA) and Fisher Scientific (Hampton, NH) unless otherwise indicated. TDP-43_102–269_ and TDP-43_1–102_ were obtained as previously described^83,84^. The TDP-43 expression strain was described previously^50,51^. The Hsc70.3-WT or Hsc70.3^K97S^ *Drosophila* strains were obtained from the Bloomington *Drosophila* stock center, Indiana, USA. All fly experiments were carried out at 25 °C in standard cornmeal molasses agar.

### Plasmids

All BioID plasmids were made using In-Fusion Recombination. mycBioID pBabe (Addgene #80901) was used as the control plasmid. TDP-43 was amplified via PCR from a pDuet TDP43 WT (purchased from Addgene, Plasmid #27462) with an AgeI restriction enzyme (RE) site built into the 5’ primer upstream of TDP-43. Amplified PCR product was inserted into mycBioID pBabe (Addgene #80901), using XhoI and SalI RE sites. The SV40 nuclear localization signal (NLS–PKKKRKV) was inserted in tandem (3x) into the newly made BioID2-TDP43 pBabe using XhoI and AgeI. Similarly, the classic protein kinase inhibitor nuclear export signal (NES- NELALKLAGLDI) was inserted into BioID2-TDP43 pBabe using XhoI and AgeI.

### Methods

#### Hsp70 isoforms purification

BL21-Codon plus bacteria (Agilent) were transformed with pSpeedET vectors containing Hsp70 isoform. Cells were grown to and OD_600_ of 0.6 at 37 °C before being shifted to 16 °C. Expression was induced once the OD_600_ reached 0.8–1.0 with 0.5 M IPTG overnight. Cells were then harvested, lysed, and protein was purified using cobalt IMAC resin (Gold bio). The His_6_ tag was cleaved using TEV protease overnight in dialysis into buffer A (50 mM HEPES pH 7.4, 100 mM KCl, 10 mM Mg(OAc)_2_, and 1 mM DTT). After complete cleavage, the DTT was dialyzed out for 4 h, and the TEV protease was recaptured with cobalt resin. Protein was then concentrated, flash frozen on liquid nitrogen and stored at − 80 °C.

#### Microscale thermophoresis

Purified TDP43_102-269_-His was labelled using the Monolith Protein Labeling Kit RED-NTA (Nanotemper, Germany) according to the manufacturer’s instructions. Briefly, 50 nM of labeled protein was mixed with ranging concentration of Hsp70 isoforms in MST buffer. The thermographs were recorded using MST premium capillaries at 40% LED and medium MST power. Data analysis was performed with the MO Affinity Analysis software (Nanotemper).

#### Synthesis and blotting of SPOT membranes

Peptides of TDP-43 (15 amino acids in length) were spotted on nitrocellulose on glass slides. Peptides were synthesized using standard 9-fluorenylmethoxycarbonyl (Fmoc) chemistry, in 30 × 20 spot arrays using a Multipeptide synthesizer adapted for SPOT synthesis (Intavis AG, Cologne, Germany). Membranes were blocked for at least 1 h in Tris-buffered saline containing 0.5% Tween 20 (TBST) with 5% semi-skimmed milk powder before an overnight incubation with 2.5 μM of HspA5 and 1 mM ADP at 4 °C with gentle shaking. Following a series of washes in TBST, the blot was probed for an hour with an HspA5 antibody at 4 °C. The following day, blots were washed three times for 10 min each time in TBST, incubated in secondary antibody (IgG (H + L) Cross Adsorbed Secondary Antibody, DyLight 800 (ThermoFisher, Product # SA5-10176) at dilutions 1:5000) for 45 min at room temperature, and washed in TBST three more times for 10 min each time before visualizing SPOTs by exposing the membranes.

#### Drosophila stocks and maintenance

The full genotypes and source of all Drosophila stocks are described in Table S2, and the genotypes represented in Fig. 4 and Fig. S3 are described in Table S3. Briefly, transgenic lines for TDP-43 and LacZ were described previously^50,51^. The UAS-Hsc70.3WT, UAS-Hsc70.3^K97S^, UAS-Hsc70.3^D231S^, si.Hsc70.3 and si.mCherry lines were obtained from the Bloomington Drosophila stock center, Indiana, USA. All Drosophila experiments were carried out at 25 °C on Bloomington cornmeal food.

#### External eye microscopy, paraffin sectioning and quantification

For external eye imaging, female Drosophila were imaged with a Leica Z16 Apo A microscope, DFC420 camera and 2.0 × planapochromatic objective. For paraffin sections, Drosophila heads were fixed, processed and quantified as previously described^85^. Eight micrometers paraffin sections were cut and mounted onto glass slides. Three sections per head were imaged at the same anatomical position and the retinal width and vacuolization was quantified using image J software. Graphpad 6 was used to determine statistical significance.

#### Drosophila immunoblotting

Immunoblotting was performed as previously described^57^. Briefly, TDP-43 or LacZ was expressed in the eye with gmr-GAL4, protein was extracted from 5 to 10 male (TDP-43) or female (LacZ) heads in 10 μl/head of 2X Laemelli buffer with 5% (v/v) β-mercaptoethanol, denatured at 95 °C, chilled on ice for 5 min and centrifuged at 5000 rpm for 5 min at 4 °C. Half a fly head (5 μL) was electrophoresed on a 4–12% bis–tris gel and transferred onto nitrocellulose by wet transfer (30 V for 65 min). Blots were blocked in 5% milk in TBST (TBS supplemented with 0.05% TWEEN-20, pH 8). Primary antibodies made up in TBST were: TDP-43 (1 in 10,000; Proteintech, #10782-2AP), α-Tubulin-HRP (1 in 5,000; Cell Signaling Technology, #9099) and β-galactosidase (1 in 15,000; Promega, #Z3781). Horseradish peroxidase (HRP)-coupled secondary antibodies made up in TBST were goat anti-rabbit-HRP (1 in 5,000; EMD Millipore #AP307P) and goat anti-mouse-HRP (1 in 10,000; abcam, ab6789). All experiments were carried out on three or more biological replicates, blots were quantified with ImageJ^86^ and statistical analysis was carried out using Graphpad prism 6 software.

#### Drosophila real time PCR

RNA was prepared from ~ 50 Drosophila heads as previously described^57^. Briefly, heads were homogenized in 1 ml of Trizol (ThermoFisher). After adding 200 µL of chloroform (Thermo Scientific), the tube was shaken for 15 s, centrifuged for 10 min at 4 °C, and the aqueous phase was transferred to a fresh tube. RNA was precipitated in ethanol and 3 M sodium acetate pH 5.2 (ThermoFisher) on ice for 25 min. Samples were centrifuged at maximum speed at 4 °C for 30 min. The RNA pellet was washed in 70% ethanol and centrifuged at maximum speed at 4 °C for 15 min. The pellet was dissolved in RNase-free water (ThermoFisher). Genomic DNA was digested with DNA-free DNase (ThermoFisher). First-strand DNA was synthesized using 300 ng of RNA and Superscript III (ThermoFisher) and random primers. Luna Universal qPCR Master Mix (NEB) was used for real-time PCR analysis. Standard curves were performed to test primer efficiency. Each experiment was carried on 3 independent fly crosses each with 3 technical repeats. Statistics were calculated using Graphpad prism 9 software. Primers to Hsc70.3 designed by the fly primer bank were used (https://www.flyrnai.org/flyprimerbankused). Primers were:

Hsc70.3 Fw: 5’ GATTTGGGCACCACGTATTCC 3’.

Hsc70.3 Rv: 5’GGAGTGATGCGGTTACCCTG 3’.

α-Tubulin Fw: 5’ CATCCAAGCTGGTCAGTG 3’.

α-Tubulin Rv: 5’ GCCATGCTCATCGGAGAT 3’.

#### Cell culture

SH-SY5Y cells were obtained from the American Type Culture Collection (ATCC; CCL-2266™). BioID stable cell lines for were generated using retroviral transduction. HEK293 Phoenix cells (National Gene Vector Biorepository, Indianapolis, IN) were transfected with each construct using Lipofectamine 3000 (Thermo Fisher Scientific) per manufacturer's recommendation. The transfected cells were incubated at 37 °C for 6 h. After 6 h incubation, the transfected cells were replenished with fresh medium and further incubated at 32 °C for 72 h. The culture media was filtered through a 0.45-μm filter and added to SH-SY5Y cells along with Polybrene (4 μg/mL; Santa Cruz Biotechnology, Dallas, TX). At 72 h after transduction, puromycin (2.5 μg/mL; Thermo Fisher Scientific) was added to the target cells. Stable cells lines were verified for fusion-protein expression and proper localization using immunofluorescence and western blot. The stable cell lines were maintained in 5.0% CO_2_ at 37 °C in DMEM/F12 1:1 (HyClone, Logan, UT) supplemented with 10% fetal bovine serum. All cells were tested monthly for mycoplasma contamination.

#### Immunofluorescence

Cells grown on glass coverslips were fixed in 3% (wt/vol) paraformaldehyde/phosphate-buffered saline (PBS) for 10 min and permeabilized by 0.4% (wt/vol) Triton X-100/PBS for 15 min. For labeling fusion proteins, a chicken anti-BioID2 antibody was used (1:5000; BID2-CP-100; BioFront Technologies). The primary antibody was detected using Alexa Fluor 568–conjugated goat anti-chicken (1:1000; A11041; Thermo Fisher Scientific). Alexa Fluor 488–conjugated streptavidin (S32354; Thermo Fisher Scientific) was used to detect biotinylated proteins. DNA was detected with Hoechst dye 33342. Coverslips were mounted using 10% (wt/vol) Mowiol 4–88 (Polysciences). Confocal images were obtained using a Nikon A1 confocal microscope (60 × /1.49 oil APO TIRF Nikon objective) with a charge-coupled device camera (CoolSnap HQ; Photometrics) linked to a workstation running NIS-Elements software (Nikon, Melville, NY). Epifluorescence images were captured using a Nikon Eclipse NiE (20 × /0.75 Plan Apo Nikon objective) microscope.

#### Western Blot analysis

To analyze total cell lysates by immunoblot, 1.2 × 10^6^ cells were lysed in SDS–PAGE sample buffer, boiled for 5 min, and sonicated to shear DNA. Proteins were separated on 4–20% gradient gels (Mini-PROTEAN TGX; Bio-Rad, Hercules, CA) and transferred to nitrocellulose membrane (Bio-Rad). After blocking with 10% (vol/vol) adult bovine serum and 0.2% Triton X-100 in PBS for 30 min, the membrane was incubated with chicken anti-BioID2 antibody (1:5000; BID2-CP-100; BioFront Technologies) overnight, washed with PBS and detected using horseradish peroxidase (HRP)–conjugated anti-chicken (1:40,000; A9046; Sigma-Aldrich). The signals from antibodies were detected using enhanced chemiluminescence via a Bio-Rad ChemiDoc MP System (Bio-Rad, Hercules, CA). Following detection of BioID2, the membrane was quenched with 30% H_2_O_2_ for 30 min. To detect biotinylated proteins, the membrane was incubated with HRP-conjugated streptavidin (1:40,000; ab7403; Abcam) in 0.4% Triton X-100 in PBS for 45 min.

#### BioID pulldowns

Large-scale BioID pulldowns were performed as described in^56^ with four 10 cm dishes per sample instead of two. In brief, four 10 cm dishes at 80% confluency were incubated with 50 μM biotin for 18 h. Cells were lysed in 8 M urea 50 mM Tris pH 7.4 containing protease inhibitor (87785, Thermo Fisher Scientific) and DTT, incubated with universal nuclease (88700, Thermo Fisher Scientific), and sonicated to further shear DNA. Lysates were precleared with Gelatin Sepharose 4B beads (17095601; GE Healthcare) for 2 h and then incubated with Streptavidin Sepharose High Performance beads (17511301, GE Healthcare) overnight. Streptavidin beads were washed four times with 8 M urea 50 mM Tris pH 7.4 wash buffer and resuspended in 50 mM ammonium bicarbonate with 1 mM biotin.

#### Sample preparation for mass spectrometry

Beads were resuspended with 8 M urea, 50 mM ammonium bicarbonate, and cysteine disulfide bonds were reduced with 10 mM tris(2-carboxyethyl)phosphine (TCEP) at 30 °C for 60 min and cysteines were then alkylated with 30 mM iodoacetamide (IAA) in the dark at room temperature for 30 min. Following alkylation, urea was diluted to 1 M urea, and proteins were subjected to overnight digestion with mass spec grade Trypsin/Lys-C mix (Promega, Madison, WI). Finally, beads were pulled down and the solution with peptides collected into a new tube. Affinity purification was carried out in a Bravo AssayMap platform (Agilent) using AssayMap streptavidin cartridges (Agilent). Digested peptides were then desalted in a Bravo AssayMap platform (Agilent) using AssayMap C18 cartridges and dried down in a SpeedVac concentrator.

#### LC–MS/MS analysis

Prior to LC–MS/MS analysis, dried peptides were reconstituted with 2% ACN, 0.1% FA and concentration was determined using a NanoDrop™ spectrophometer (ThermoFisher). Samples were then analyzed by LC–MS/MS using a Proxeon EASY-nanoLC system (ThermoFisher) coupled to a Q-Exactive Plus mass spectrometer (Thermo Fisher Scientific). Peptides were separated using an analytical C18 Aurora column (75 µm × 250 mm, 1.6 µm particles; IonOpticks) at a flow rate of 300 nL/min (60 °C) using a 120-min gradient: 1% to 5% B in 1 min, 6% to 23% B in 72 min, 23% to 34% B in 45 min, and 34% to 48% B in 2 min (A = FA 0.1%; B = 80% ACN: 0.1% FA). The mass spectrometer was operated in positive data-dependent acquisition mode. MS1 spectra were measured in the Orbitrap in a mass-to-charge (*m/z*) of 350–1700 with a resolution of 70,000 at *m/z* 400. Automatic gain control target was set to 1 × 10^6^ with a maximum injection time of 100 ms. Up to 12 MS2 spectra per duty cycle were triggered, fragmented by HCD, and acquired with a resolution of 17,500 and an AGC target of 5 × 10^4^, an isolation window of 1.6 m/z and a normalized collision energy of 25. The dynamic exclusion was set to 20 s with a 10 ppm mass tolerance around the precursor.

#### MS data analysis

All mass spectra were analyzed with MaxQuant software version 1.6.11.0. MS/MS spectra were searched against the *Homo sapiens* Uniprot protein sequence database (downloaded in January 2020) and GPM cRAP sequences (commonly known protein contaminants). Precursor mass tolerance was set to 20 ppm and 4.5 ppm for the first search where initial mass recalibration was completed and for the main search, respectively. Product ions were searched with a mass tolerance 0.5 Da. The maximum precursor ion charge state used for searching was 7. Carbamidomethylation of cysteine was searched as a fixed modification, while oxidation of methionine and acetylation of protein N-terminal were searched as variable modifications. Enzyme was set to trypsin in a specific mode and a maximum of two missed cleavages was allowed for searching. The target-decoy-based false discovery rate (FDR) filter for spectrum and protein identification was set to 1%. Interaction candidates were those proteins enriched at least 3 × over control samples (BioID2-only) and identified in at least two of the three experimental triplicate samples (N > 2).

#### Immunohistochemistry

Samples from the prefrontal cortex and spinal cord of ALS and control patients were obtained from the University of Michigan Brain Bank. Consent for autopsy was obtained in accordance with guidelines from the University of Michigan Brain Bank who reviewed and confirmed that protocols met the criteria for human-subjects research. Immunostaining was accomplished using the Dako Autostainer Link 48 (Agilent, USA). Anti-HspA5 antibody (Abcam ab21685) was used at 1:1000 with the Dako High pH Target Retrieval Solution (Tris/EDTA, pH 9; Agilent, USA) (20 min, 97°) and the Dako Envision Flex Plus Mouse Link Kit (Agilent, USA) to detect the antibody along with the Dako DAB (Agilent, USA). The images were analyzed using free, open-access QuPath (v.0.3.2) software and were analyzed by a blinded experimentator.

## Supplementary Information

Supplementary Information 1.

Supplementary Table S1.
