# Supplementary material for: Heat shock protein Grp78/BiP/HspA5 binds directly to TDP-43 and mitigates toxicity associated with disease pathology
Source: Sci Rep. 2022 May 17;12:8140. doi: 10.1038/s41598-022-12191-8 (PMC9114370; doi:10.1038/s41598-022-12191-8)
Supplement: Supplementary file 1 — Supplementary Information 1. [file 41598_2022_12191_MOESM1_ESM.docx]

**Supplementary information**

**Heat shock protein A5 directly binds to TDP-43 and mitigates toxicity associated with disease pathology.**

**Liberty François-Moutal^1,2^, David Donald Scott^1,2^, Andrew J. Ambrose^3^, Christopher J. Zerio^3^, Marina Rodriguez-Sanchez^4^, Kumara Dissanayake^5^, Danielle G. May^6^, Jacob M. Carlson^1,2^, Edward Barbieri^7^, Aubin Moutal^1,2^, Kyle J. Roux^6,8^, James Shorter^8^, Rajesh Khanna^1,2^, Sami J. Barmada^9^, Leeanne McGurk^5^ and May Khanna^1,2,4*^**

From the ^1^Department of Pharmacology, College of Medicine, University of Arizona, Tucson, AZ 85724, USA; ^2^Center for Innovation in Brain Science, Tucson, AZ 85721, USA; ^3^Pharmacology and Toxicology, School of Pharmacy, University of Arizona, Tucson, AZ 85724, USA; ^4^Department of Molecular Pathobiology, NYU, New York, NY; ^5^Cell and Developmental Biology, School of Life Sciences, University of Dundee, Dow Street, Dundee DD1 5EH, UK; ^6^Enabling Technologies Group, Sanford Research, Sioux Falls, SD; ^7^Department of Biochemistry and Biophysics, Perelman School of Medicine, University of Pennsylvania, Philadelphia, PA 19104, USA; ^8^Department of Pediatrics, Sanford School of Medicine, University of South Dakota, Sioux Falls, SD; ^9^Department of Neurology, University of Michigan, Ann Arbor, MI 48109

* To whom correspondence should be addressed: Dr. May Khanna, Department of Pharmacology, College of Medicine, University of Arizona, 1501 North Campbell Drive, P.O. Box 245050, Tucson, AZ 85724, USA Office phone: (520) 626-2147; Fax: (520) 626-2204; Email: [maykhanna@email.arizona.edu](mailto:maykhanna@email.arizona.edu).

**Supplementary tables**

***Table S2: Drosophila strains***

| ***Label*** | ***Genotype*** | ***Source*** |
| --- | --- | --- |
| normal | *y[1] sc[*] v[1] sev[21]; P{y[+t7.7] v[+t1.8]=VALIUM20-mCherry}attP2* | Bloomington, #35785 |
| TDP-43 | *UAS-TDP-43 (37M)/CyO; gmr-GAL4(YH3)/TM6B* | Elden et al 2010, Kim et al 2014 |
| Hsc70.3^WT^ | *w[126]; P{w[+mC]=UAS-Hsc70-3.WT}B; +/+* | Bloomington # 5843 |
| Hsc70.3^K97S^ | *w[126]; P{w[+mC]=UAS-Hsc70-3.K97S}D; +/+* | Bloomington # 5842 |
| LacZ | *w-;; +/+; UAS-lacZ; gmr-GAL4 (YH3)* | Elden et al 2010, Kim et al 2014 |
| Hsc70.3^D231S^ | *w[126]; +/+; P{w[+mC]=UAS-Hsc70-3.D231S}D* | Bloomington # 5841 |
| si.Hsc70.3 | \|  \| *y[1] sc[*] v[1] sev[21]; P{y[+t7.7] v[+t1.8]=TRiP.HMS00397}attP2* \| \| --- \| --- \| | Bloomington # 32402 |

***Table S3: Drosophila genotypes related to Figure 4 and Supplementary Figure 3.***

| **Figure** | **Label** | **Genotype** |
| --- | --- | --- |
| 5A,B,  C,D,E | normal | *y, sc, v, sev/w^1118^; +/+; gmr-GAL4 (YH3)/ UAS-si.mCherry^35783^* |
|  | ctrl | *y, sc, v, sev/w^1118^; UAS-TDP-43/+; gmr-GAL4 (YH3)/ UAS-si.mCherry^35783^* |
|  | Hsc70.3^WT^ | *w-; UAS-TDP-43/UAS-Hsc70-3^WT^; gmr-GAL4 (YH3)/+* |
|  | Hsc70.3^K97S^ | *w-; UAS-Hsc70.3^K97S^/+;* *gmr-GAL4 (YH3)/+* |
| 5F | ctrl | *y, sc, v, sev/w^1118^; UAS-LacZ/+; gmr-GAL4 (YH3)/ UAS-si.mCherry^35783^* |
|  | Hsc70.3^WT^ | *w-; UAS-lacZ/UAS-Hsc70-3^WT^; gmr-GAL4 (YH3)/+* |
|  | Hsc70.3^K97S^ | *w-; UAS-lacZ/UAS-Hsc70-3^K97S^; gmr-GAL4 (YH3)/+* |
| 5G | ctrl | *y, sc, v, sev/w^1118^; +/+; gmr-GAL4 (YH3)/UAS-si.mCherry^35783^* |
|  | TDP-43 | *y, sc, v, sev/w^1118^; UAS-TDP-43/+; gmr-GAL4 (YH3)/ UAS-si.mCherry^35783^* |
| S3B | normal | *y, sc, v, sev/w^1118^; +/+; gmr-GAL4 (YH3)/UAS-si.mCherry^35783^* |
|  | Hsc70.3^WT^ | *w-; UAS-TDP-43/ UAS-Hsc70-3^WT^; gmr-GAL4 (YH3)/+* |
|  | Hsc70.3^K97S^ | *w-; UAS-Hsc70-3.K97S/+; gmr-GAL4 (YH3)/+* |
|  | Hsc70.3^D231S^ | *UAS-Hsc70-3.D231S/+; gmr-GAL4 (YH3)/+* |
|  | si.Hsc70.3 | *y, sc, v, sev/w^1118^; +/+; gmr-GAL4 (YH3)/UAS-si.Hsc70.3^HMS00397^* |

***Table S4: Description of patients related to Supplementary Figure 4.***

| **Age at death** | **Sex** | **PMI (h)** | **Diagnosis** | **Section** |
| --- | --- | --- | --- | --- |
| 45 | M | 48 | ALS | Frontal cortex |
| 74 | F | 6 | Control | Frontal cortex |
| 87 | M | 9 | Control | Frontal cortex |
| 60 | F | 33 | ALS | Frontal cortex |

**Supplementary figures**


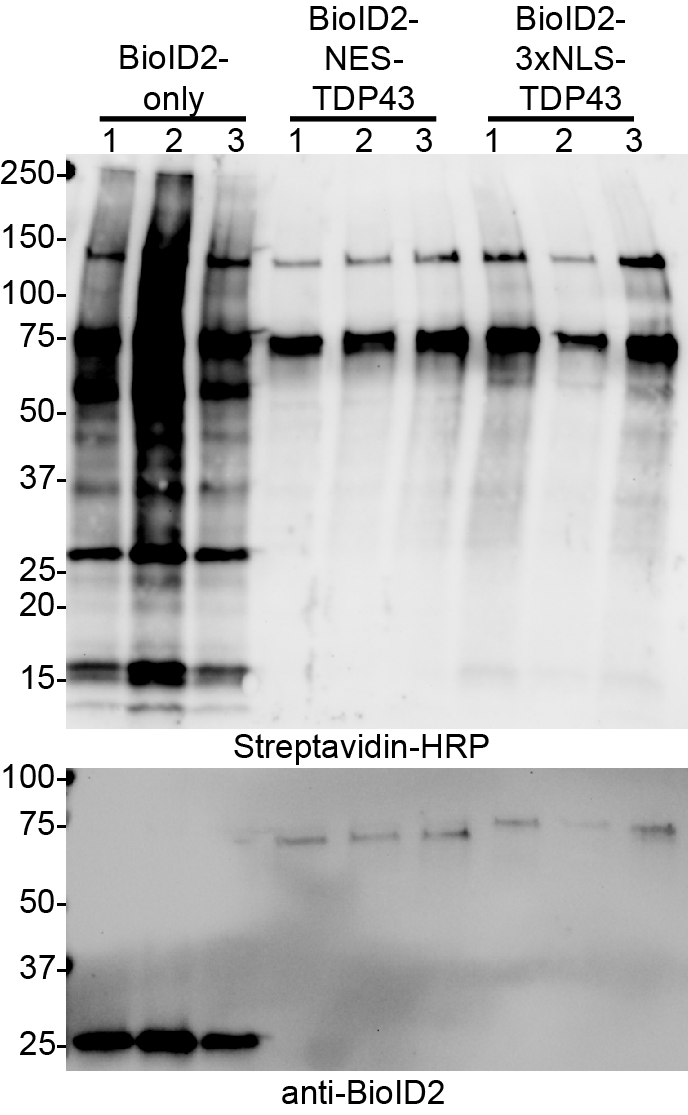


**Supplementary figure 1: BioID of TDP-43 in SHSY5Y cells in nucleus and cytoplasm.** Western blot analysis of BioID2 fusion-protein and biotinylation following BioID pulldown in triplicate.


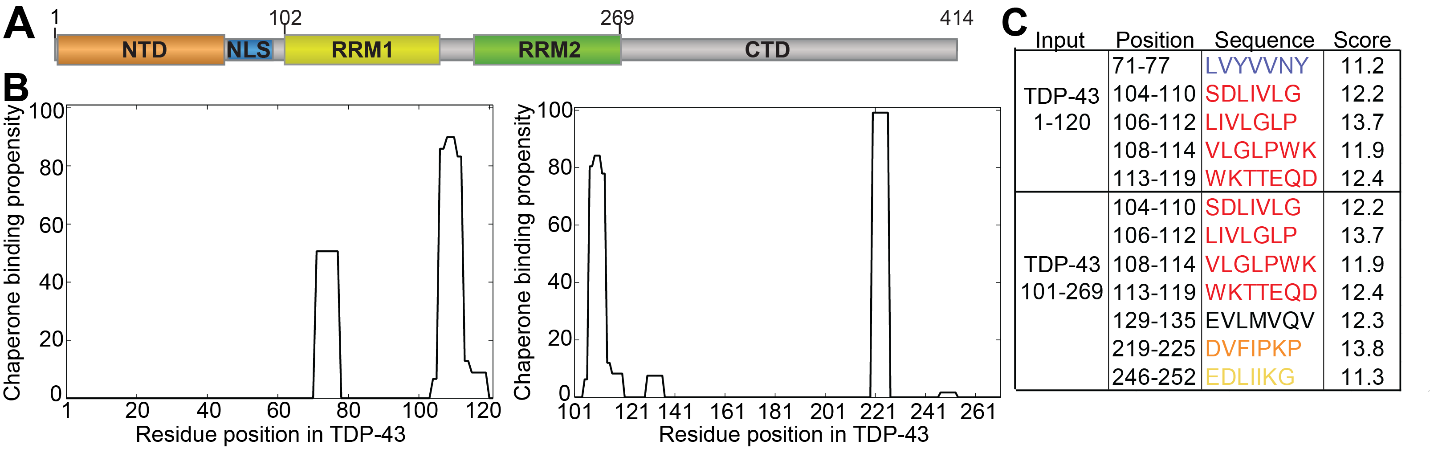


**Supplementary figure 2: Mapping of Hsp70 binding to TDP-43. A.** Primary structure of TDP-43. **B.** Chaperone binding propensities calculated by LIMBO on the different inputs aa 1-120 (N-terminal domain and flexible linker of TDP-43), aa 101-269 (the two RNA Recognition Motifs). **C** Client peptide sequences and positions in TDP-43 were scored by LIMBO. No peptide was predicted to bind the C-terminus region of TDP-43 (input: aa. 270-414).

**
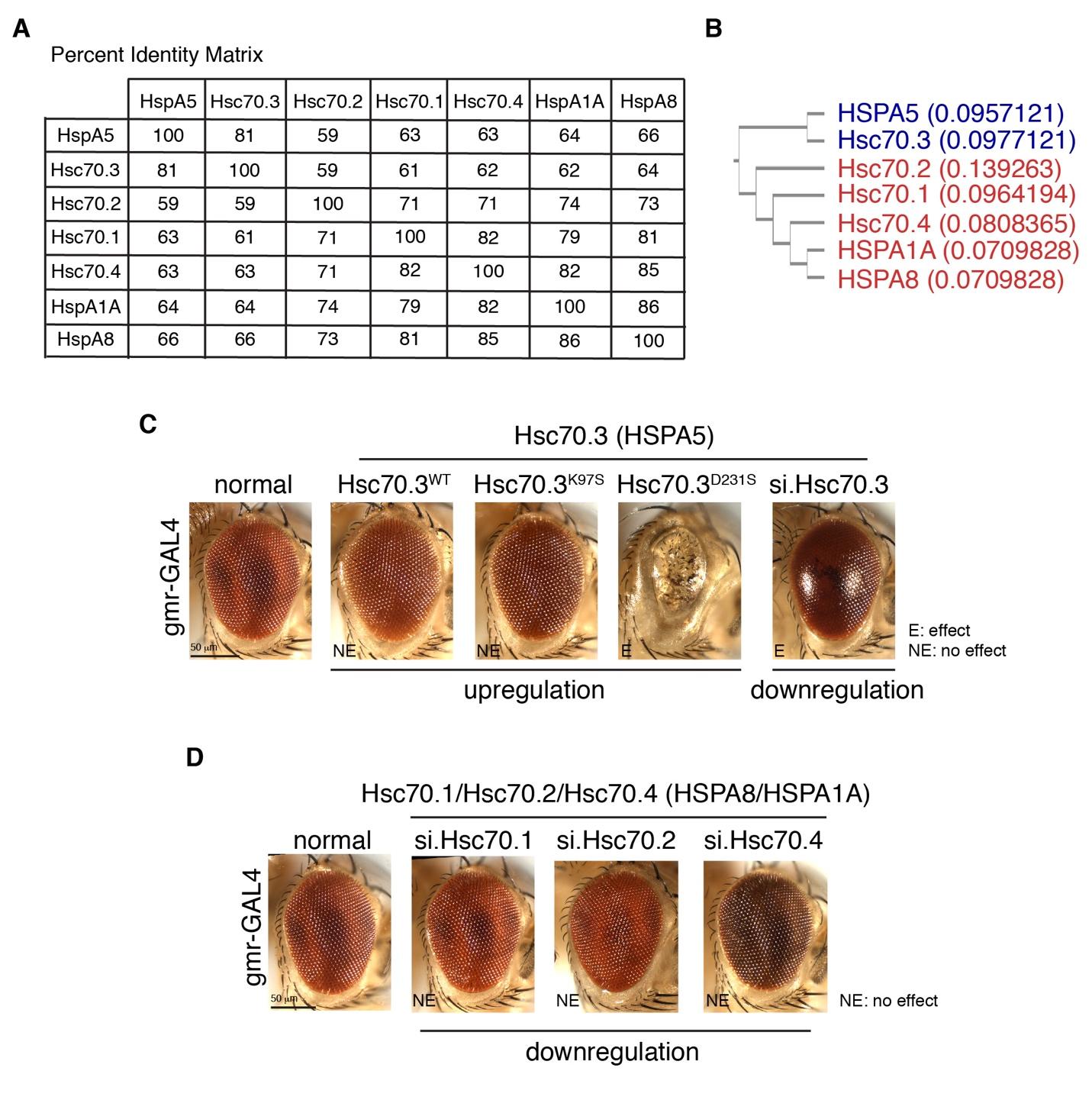
**

**Supplementary figure 3: Selection of HSPA5, HSPA8, and HSPA1A homologues in *Drosophila*. A.** The human protein sequence for HSPA5, HSPA1A and HSPA8 were blasted against the Drosophila proteome, which resulted in a >60 entries for each of the proteins. To identify the homologues entries were stratified first on sequence identify and secondly on coverage of the full sequence of the human protein, thus ensuring that the signal peptides at the N- and C-termini were present. This revealed that the fly gene Hsc70-3 is the homologue to HSPA5A. Hsc70.1, Hsc70.2 and Hsc70.4 aligned to both HSPA8 and HSPA1A with high sequence identity. B. A phylogenetic tree representing the alignment of HSPA5, HSPA8, HSPA1A, Hsc70.1, Hsc70.2, Hsc70.3 and Hsc70.4 (used simple phylogeny, EMBL-EBI. Parameters: NEXUS, UPGMA). **C.** *Drosophila* strains that upregulate or downregulate Hsc70.3 were mated to the *gmr*-GAL4 driver line to test for an effect on eye structure compared to the normal eye. This revealed that expression of the wild type form of Hsc70.3 (UAS-Hsc70.3^WT^) or a form of Hsc70.3 defective in ATP binding (Hsc70.3^K97S^) had no effect on the Drosophila eye. By contrast expression of Hsc70.3 harbouring the D231S mutation, which also inhibits ATP binding severely disrupted the *Drosophila* eye.

Genotypes

**C**: normal is y*, sc, v, sev/w^1118^; +/+; gmr-GAL4 (YH3)/si.mCherry^35783^*, Hsc70.3^WT^ is *w-; UAS-Hsc70-3.WT/+; gmr-GAL4 (YH3)/+,* Hsc70.3^K97S^ is *w-; UAS-Hsc70-3.K97S/+; gmr-GAL4 (YH3)/+* and Hsc70^D231S^ is *UAS-Hsc70-3.D231S/+; gmr-GAL4 (YH3)/+*. **D**: normal is y*, sc, v, sev/w^1118^; +/+; gmr-GAL4 (YH3)/si.mCherry^35783^,* si.Hsc70.1 is *y, sc, v, sev/w^1118^; +/+; gmr-GAL4 (YH3)/si.Hsc70.1^HMS00888^,* si.Hsc70.2 is *y, sc, v, sev/w^1118^; +/+; gmr-GAL4 (YH3)/si.Hsc70.2^44485^,* and si.Hsc70.4 is *y, sc, v, sev/w^1118^; +/+; gmr-GAL4 (YH3)/si.Hsc70.1* *^JF03136^*


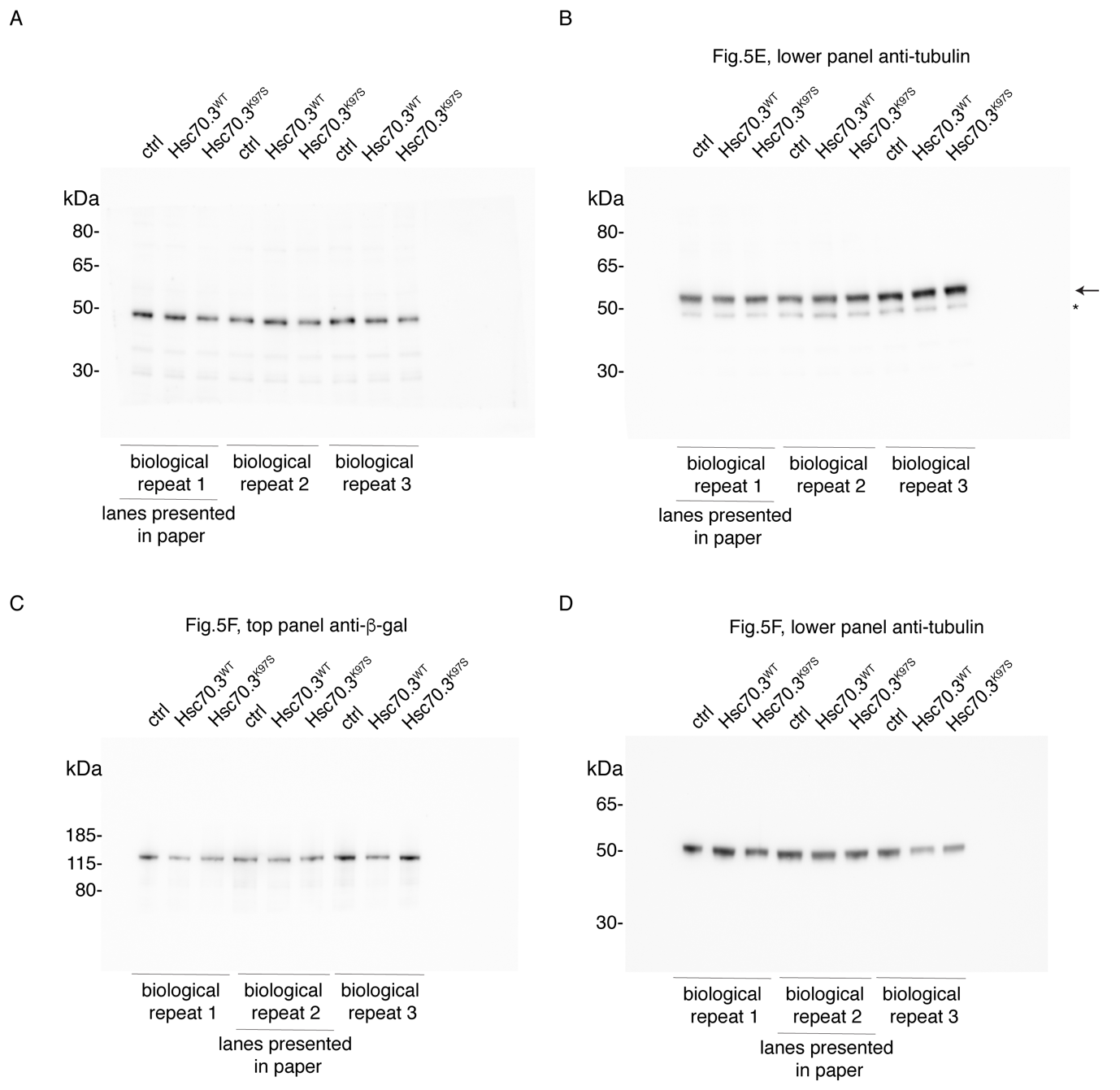


**Supplementary figure 4**: Raw and expanded immunoblots presented in Figure 4. **A.** TDP-43 Protein levels in control vs upregulation of Hsc70.3^WT^ or Hsc70.3^K97S^. Protein isolated from ~5-10 male heads immunoblotted for TDP-43 from 3 biological repeats. Lanes presented in Fig 4E are indicated. **B.** Tubulin protein levels in control vs upregulation of Hsc70.3^WT^ or Hsc70.3^K97S^. Protein isolated from ~5-10 male heads immunoblotted for TDP-43 from 3 biological repeats. Arrow indicates Tubulin band and asterisk is residual TDP-43 signal. Lanes presented in Fig 4E are indicated. **C.** Protein levels of β-galactosidase in control vs upregulation of Hsc70.3^WT^ or Hsc70.3^K97S^. Protein isolated from ~5-10 male heads immunoblotted for β-galactosidase from 3 biological repeats. Lanes presented in Fig 4F are indicated. **D.** Protein levels of Tubulin in control vs upregulation of Hsc70.3^WT^ or Hsc70.3^K97S^. Protein isolated from ~5-10 male heads immunoblotted for Tubulin from 3 biological repeats. Lanes presented in Fig 4F are indicated.


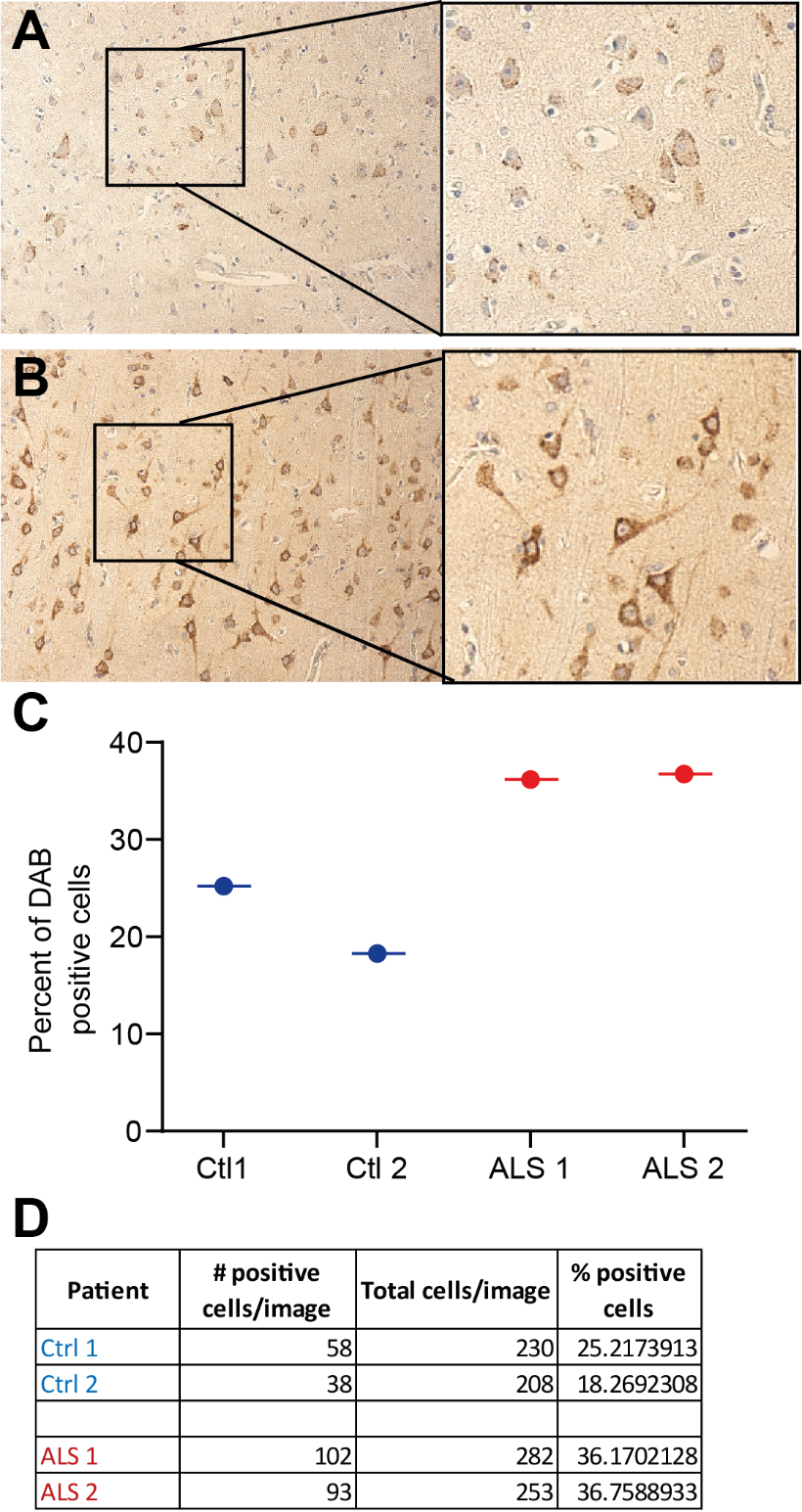


**Supplementary figure 5**:  **HspA5 is overexpressed in the cytoplasm of neurons from prefrontal cortex of ALS patients.** Deep (layer V) neurons of the frontal cortex were immunostained for HspA5, and counterstained with hematoxylin. **A.** ALS patients; **B.** control patients. Right panels are zoomed in version of left panels. **C.** Percent of positive cells was normalized based on the number of nuclei. **D.** Table showing the number of DAB positive cells, the number of nuclei and the percent of DAB positive cells.
